# Total hip arthroplasty versus hemiarthroplasty in the treatment of active elderly patients over 75 years with displaced femoral neck fractures: a retrospective study

**DOI:** 10.1186/s12891-023-06860-6

**Published:** 2023-09-20

**Authors:** Santong Luo, Wupeng Qin, Lizhi Yu, Raoshan Luo, Weiming Liang

**Affiliations:** The First Affiliated Hospital of Guangxi University of Science and Technology, Guangxi University of Science and Technology, 124 Yuejin Road, Liuzhou, 545001 Guangxi Province China

**Keywords:** Displaced femoral neck fracture, Total hip arthroplasty, Hemiarthroplasty

## Abstract

**Background:**

Femoral neck fractures are associated with substantial morbidity and mortality for older adults. Total hip arthroplasty (THA) and hemiarthroplasty (HA) are widely used in elderly patients with displaced femoral neck fractures (DFNF), but there is still controversy refering to the optimal chose for the management of DFNF in active elderly patients.

**Methods:**

This is a retrospective cohort study that incorporates medical record review with an outcomes management database. 73 patients who underwent HA and 66 patients who underwent THA were identified from January 2015 to December 2017. Data of age, gender, BMI, comorbidity status, operation time, blood loss, hospitalization time, in-hospital complication were collected and analyzed. Clinical follow-up and radiographic examinations were performed at approximately five years, and hip complications, Harris Hip Score (HHS) and EuroQol-5 Dimensions (EQ-5D) were assessed.

**Results:**

Preoperative general data of sex, age, BMI and charlson comorbidity score of THA group(*n*=55) has no statistically significant difference with that of HA group. Patients treated by THA had significantly longer operation time (105.5 vs 76.7 minutes; *P < *0.001), more blood loss (524.1 vs 350.1 ml; *P < *0.001) and longer hospitalization time (15.8 vs 13.8 days; *P* < 0.001). There was no significant differences between two groups in complications (32.7% vs 25.8%, *P*=0.432). No patients died during the hospitalization. After five years, only 33 patients in the THA group and 34 patents in the HA group were still alive, and the fraction surviving were not statistically significant between two groups (60.0% vs 54.8%, *P*> 0.05). The differences in hip function in favor of THA appeared to increase after the five-year follow-up, and the difference was significant in terms of the total Harris hip score (81.3 vs 73.1, *P* < 0.001) as well as in the dimensions of pain (38.9 vs 35.9, *P*=0.033), function (33.7 vs 29.2, *P*=0.001), absence of deformity (4.0 vs 3.9, *P*=0.023) and range of motion (4.6 vs 4.2, *P*=0.008). There was no significant differences between groups in hip dislocation rate (6.1% vs 0.0%, *P*=0.239). The erosion rate of hip joint in the THA group was significantly lower than that of the HA group (0.0% vs 26.5%, *P*=0.002). The health-related quality of life, according to EQ-5D index score, was found to be higher (0.69 vs 0.63, *P*= 0.001) in the THA group than the HA group after five years.

**Conclusion:**

THA may be a preferred management option for active elderly patients over 75 years. The more extensive surgery of THA is not associated with higher in-hospital complication rate or mortality rate. These patients can benefit from THA in terms of hip function and quality of life.

**Trial registration:**

No.

## Introduction

Femoral neck fracture(FNF) is a worldwide health problem which can cause significant morbidity and mortality [1]. Most femoral neck fractures are associated with a fall, although other risk factors include osteoporosis, chronic medication use and reduced level of activity [2, 3]. Low bone mineral density and elevated bone turnover markers are independent predictors of hip fracture risk, and the risk is multiplied when both are present [4]. Since femoral neck is primarily composed of cortical bone and requires greater proportionate changes than trabecular bone, both increase in bone mineral density and reduction in bone turnover may be necessary to decrease the risk FNF [5]. In recent years, the prevalence of FNF has increased with the acceleration of population aging, and the risk of fracture doubles every decade after age 50 [6]. Most important of all, the mortality rate is high, with the 30-day mortality of 3–10%, and the 1-year mortality rate up to 30% [7–9]. A fourth of patients with FNF require long term nursing home care before they had the ability to live independently, and half of the patients were unable to regain pre-fracture mobility [10].

The treatment for FNF includes internal fixation, total hip arthroplasty (THA) and hemiarthroplasty (HA) [11]. For young patients or the elderly intolerant of prosthesis surgery, internal fixation is a preferred management option, but THA and HA are widely used in elderly patients with displaced femoral neck fractures (DFNF) [12, 13]. HA is associated with less blood loss, shorter operation time, less economic burden, less technical demand, and a lower dislocation rate, while the advantages of THA includes better hip function, less acetabulum erosion, and a lower revision rate [14–19].

There is still controversy refer to the optimal chose for the management of DFNF in active elderly patients. Since the advantages of less pain and lower revision rate, current guidelines suggested that THA might be a preferred management option in selected elderly patients who were able to walk independently and medically fit for the procedure [20, 21]. However, a systematic review indicated that there was no significant differences in hip function, reoperation rate, and mortality rate between HA and THA [17].

In the present study, we aimed to compare the efficacy and safety of HA and THA in the treatment of DFNF in the active elderly over 75 years by a retrospective study.

## Methods

### Study design

This is a retrospective and non-randomized study that incorporates medical record review with an outcomes management database. Information for this database was collected on all active elderly over 75 years with DFNF who complied with the inclusion criteria from January 2015 to December 2017.

### Ethical approval and consent

The study was conducted according to the Declaration of Helsinki and the International Conference on Harmonisation Tripartite Guideline on Good Clinical Practice. All patients provided written informed consent before participating. Approvals from Ethics Committee of the First Affiliated Hospital of Guangxi University of Science and Technology were obtained in January 2021(approval number:2021-LC039).

### Patients

In the present study, active elderly patients were defined as patients who were able to walk independently before FNF, with no or minimal osteoarthritis [22]. Inclusion criteria: displaced femoral neck fractures, age over 75 years, independent walking prior to the injury, and no contraindication to anesthesia. Exclusion criteria: age over 90 years, old fracture that occurs more than 6 weeks ago, cancer, osteoarthritis or rheumatoid arthritis in the fractured hip, pathological fracture, and significant senile dementia. From the electronic database of our hospital, 73 patients who underwent HA and 66 patients who underwent THA were identified from January 2015 to December 2017.

### Minimum sample size for effective evaluation

A power analysis, based on a previous study with the same inclusion criteria [23], indicated that a minimum sample size of 120 patients would provide a power of 90% to identify a 5-point difference in the Harris hip score.

### Procedure of HA and THA

In all patients, Radiographs of the pelvis with bilateral hips, Radiographs of the chest, blood routine, coagulation function, biochemical indicators (including of liver function, renal function, myocardial enzyme, electrolyte, blood gas analysis), induced flow rate and cardiopulmonary function and bilateral limb vascular ultrasound examination were performed preoperatively. Prophylactic antibiotics were applied.

In all patients, the surgeons were performed via a transgluteal lateral approach, and a cemented collarless polished tapered (Dragonbio; Wuhan, China) femoral component was implanted. In the THA group, a appropriately sized cobalt chrome femoral head articulating with an all-polyethylene cemented acetabular component without a long posterior wall (Dragonbio; Wuhan, China) was implanted. In the HA group, an appropriately sized Endo Femoral Head (Dragonbio; Wuhan, China) was implanted. In both group, head size was measured intra-operatively from hemispherical templates and available in 2 mm increments. Both surgical procedures were performed by the same orthopedic surgeons.

We previously reported post-operative management [19]. After the surgeons, rehabilitation medicine doctors guide the patient to exercise muscle strength and joint mobility, and guide the patient to get early out of bed. Isometric quadriceps contraction and ankle pump training began 6 h after surgery, knee flexion and straight leg elevation started 1 day after surgery, and walking training with the help of the walker started 2 days after surgery. All patients were taking antiresorptive treatments by oral bisphosphonates.

### Data collected

Demographic data, including name, date of birth, BMI and gender were collected from the electronic database of our hospital. In patient charts (including admission notes, progress notes, operative dictations and discharge summaries) were reviewed to collect the date of admission, date and time of surgery, date of discharge, type of surgery, comorbid diagnoses and complications. Charlson Comorbidity Index [24] was used to quantify patient comorbidity. Follow-up clinical and radiographic examinations were performed at approximately five years. Numbers of patients alive at five years in both group were collected and the 5-year survival rate was assessed.

Acetabular erosion was assessed according to the latest radiographs of the pelvis with bilateral hips, using the four grades described by Baker RP et al. [25]: grade 0 (no erosion), grade 1 (narrowing of articular cartilage, no bone erosion), grade 2 (acetabular bone erosion and early migration), and grade 3 (protrusio acetabuli). The erosion rate was defined as the proportion of patients who had Grade ≥ 1 erosion to the total.

Hip complications, hip function were assessed according to the Harris hip score [26], in which the domains covered are pain, function, absence of deformity, and range of motion. The pain domain measures pain severity and its effect on activities and need for pain medication. The function domain consists of daily activities (stair use, using public transportation, sitting, and managing shoes and socks) and gait (limp, support needed, and walking distance). Deformity takes into account hip flflexion, adduction, internal rotation, and extremity length discrepancy. Range of motion measures hip flflexion, abduction, external and internal rotation, and adduction.

The health-related quality of life were assessed according to the EuroQol- 5 Dimensions (EQ-5D) [27]. The domains covered are mobility, self-care, usual activities, pain/discomfort and anxiety/depression.

### Statistical analysis

The Statistical Package for the Social Science (SPSS, version 26.0) was used for statistical analysis of the data. Numerical variables are expressed as mean ± standard deviation. Continuous variables were compared via the unpaired t test. Nominal variables were tested with Pearson’s χ2 test. Differences was considered significant at *P* < 0.05.

## Results

Characteristics of the two groups at baseline are given in Table 1. In the two groups, the comparative differences in preoperative general data of sex, age, BMI and Charlson comorbidity score were not statistically significant (P > 0.05).

**Table 1:** Characteristics of patients at baseline

Characteristic	THA(*n* = 55)	HA(*n* = 62)	*P* value
Age (mean ± SD, year)	82.8 ± 4.3	83.4 ± 5.2	0.498
Male(n,%)	30,54.4%	26,41.9%	0.280
BMI ( mean ± SD)	24.5 ± 3.6	23.8 ± 3.5	0.076
Charlson comorbidity score ( mean ± SD)	2.5 ± 1.2	2.6 ± 1.3	0.987

Operative details of the patients are given in Table 2. Patients treated by THA had significantly longer operation time (105.5 vs. 76.7 min; *P* <0.001), more blood loss (524.1 vs. 350.1ml; *P* <0.001) and longer hospitalization time (15.8 vs. 13.8 days; *P* <0.001).

**Table 2:** Operative details of the patients

	THA(*n* = 55)	HA(*n* = 62)	*P* value
Operation time(mean ± SD, min)	105.0 ± 14.8	76.7 ± 13.4	< 0.001
Blood loss ( mean ± SD, ml)	524.1 ± 171.0	350.1 ± 82.2	< 0.001
Hospitalization time (mean ± SD, day)	15.8 ± 3.2	13.8 ± 2.7	< 0.001

The data in Table 3 shows the differences with respect to in-hospital complication between the two groups. There was no significant differences between two groups in complications overall. With respect to delirium, postoperative infection, renal insufficiency, gastrointestinal tract bleeding, cardiac, hypoxia, thromboembolism or stroke, there was also no significant differences between two groups. No patients died during the hospitalization.

**Table 3:** In-hospital complication of patients

	THA(*n* = 55)	HA(*n* = 62)	*P* value
Complications overall(n, %)	18,32.7%	16,25.8%	0.423
Delirium (n, %)	7,12.7%	6,9.7%	0.600
Postoperative infection(n, %)	2,3.6%	1,1.6%	0.489
Renal insufficiency(n, %)	1,1.8%	0,0.0%	0.286
Gastrointestinal tract bleeding(n, %)	2,3,6%	0,0.0%	0.130
Cardiac(n, %)	4,7.3%	3,4.8%	0.580
Hypoxia(n, %)	5,9.1%	7,11.3%	0.457
Thromboembolism(n, %)	3,5.5%	2,3.2%	0.552
Stroke(n, %)	1,1.8%	0,0.0%	0.286
Death(n, %)	0,0.0%	0,0.0%	1

The data in Table 4 shows the outcome measured at 5-year follow-up. After five years, only 33 patients in the THA group and 34 patents in the HA group were still alive, and the fraction surviving were not statistically significant between two groups (60.0% vs. 54.8%, P > 0.05). The differences in hip function in favor of THA appeared to increase after the five-year follow-up, and the difference was significant in terms of the total Harris hip score (81.3 vs. 73.1, P<0.001) as well as in the dimensions of pain (38.9 vs. 35.9, *P* = 0.033), function (33.7 vs. 29.2, *P* = 0.001), absence of deformity (4.0 vs. 3.9, *P* = 0.023) and range of motion (4.6 vs. 4.2, *P* = 0.008). There was no significant difference between two groups in hip dislocation rate (6.1% vs. 0.0%, *P* = 0.239). The erosion rate of hip joint in the THA group was significantly lower than that of the HA group(0.0% vs. 26.5%, *P* = 0.002). The health-related quality of life, according to EQ-5D index score, was found to be higher (0.69 vs. 0.63, *P* = 0.001) in the THA group than the HA group after five years.

**Table 4:** Outcome measures at 5-year follow-up

	THA(*n* = 33)	HA(*n* = 34)	*P* value
Fraction surviving(n, %)	33,60.0%	34,54.8%	0.573
Total Harris Hip Score( mean ± SD)	81.3 ± 6.5	73.1 ± 10.6	< 0.001
Pain( mean ± SD)	38.9 ± 4.5	35.9 ± 6.7	0.033
Function ( mean ± SD)	33.7 ± 4.6	29.2 ± 6.5	0.001
Absence of deformity( mean ± SD)	4.00 ± 0.00	3.85 ± 0.36	0.023
Range of motion( mean ± SD)	4.64 ± 0.49	4.24 ± 0.70	0.008
EQ-5D( mean ± SD)	0.69 ± 0.08	0.63 ± 0.08	0.001
Hip dislocation (n, %)	2,6.1%	0,0.0%	0.239
Erosion(n, %)	0,0.0%	9,26.5%	0.002

## Discussion

The optimal chose for the management of DFNF in active elderly patients remains controversy [17]. In the present study, the efficacy and safety of HA and THA in the treatment of DFNF in the active elderly over 75 years was compared by a retrospective study. Our study shows that the THA group experience better outcomes than those in the HA group. Specifically, patients treated by THA had shorter operation time, more blood loss and longer hospitalization time, but there was no significant differences between two groups in in-hospital complications. At 5-year follow-up, the fraction surviving were not statistically significant between two groups, but the THA group has a better functional results and better health-related quality of life than the HA group. There was no significant differences between groups in hip dislocation rate, while the HA group has a higher erosion rate.

Since additional acetabular reconstruction is required, the THA procedure is usually associated with longer operation time and more blood loss. In the present study, THA caused an extra half an hour of operation time and about 200ml of bleeding compared with HA. These findings corroborate with the results of previous literature on the topic conducted in the past [28, 29].

Our study included elderly patients aged over 75 years who had relatively low tolerance of surgery, so perioperative complications are a very important concern. Though an increased number of hip complications occurred in the total hip arthroplasty group in the present study, the difference was not significant. Similar with our results, previous studies had also shown no significant difference between THA and HA [30, 31]. However, other studies showed higher general complication rates by THA [29]. Therefore, more clinical data are needed to further confirm this issue. On the other hand, as Hedbeck CJ et al. had declared, even if there is a minor increase in the risk of hip complications after total hip arthroplasty as compared with hemiarthroplasty, the risk-taking may be justified on the basis of the better hip function after total hip arthroplasty [30].

Previous studies reported different outcomes between THA and HA refer to dislocation. Some randomized controlled trial found no significant difference in dislocation rate between two procedures in patients over 75 years [30, 32], but other studies found a higher dislocation rate in the THA group [16, 17, 29]. In the present study, though two patients in THA group but no patient in HA group had a dislocation. There was no significant differences between groups in hip dislocation rate. The two patients with dislocations were managed conservatively without revision surgery. Compared with previous study [17], our result showed a lower trend of dislocation rate in patients over 75 years, which may be caused by the lower level of activity. Acetabular erosion is a common long-term complication of HA which would lead to painful symptoms for patients despite the lower physical demand. Consistent with our results, previous studies reported significant higher erosion rate in the HA group than that of THA [29, 32].

Harris hip score has been used in previous studies for recording the outcome after a fracture of the femoral neck [15, 32]. Whether the superior hip function of THA in this population still exists in the long term is controversial. Some studies reported better total HHS outcomes [25, 29], while other studies showd no significant difference in total HHS and pain HHS in elderly patients [17, 32]. Our results showed a better score at 5-year follow-up in the THA group in total HHS, as well as in the dimensions of pain, function, absence of deformity and range of motion. The health-related quality of life were assessed according to the EQ-5D, and patients treated by THA was found to have a better outcome, indicating that the improvement of hip function by.

THA could significantly improve the quality of life.

In addition, due to lack of health education, most patients treated in our hospital have not known osteoporosis before FNF. After THA or HA, all patients were suggested to take antiresorptive treatments by oral bisphosphonates. According to Postmenopausal Osteoporosis Guidelines [33], pharmacologic therapy is strongly recommended for patients with osteopenia or low bone mass and a history of fragility fracture of the hip or spine. Approved agents with efficacy to reduce hip, nonvertebral, and spine fractures including alendronate, denosumab, risedronate, and zoledronate are appropriate as initial therapy for most osteoporotic patients with high fracture risk.

The present study had several strengths. First of all, controversy still remains in terms of the optimal chose for the management of DFNF in active elderly patients, and our results provides available evidence that THA has advantage over HA in the treatment of active elderly patients over 75 years with DFNF. Second, with a follow-up of > five years, our study provides long-term outcomes of elderly patients treated by THA or HA. However, there were several limitations in our study. First of all, since our study is a retrospective study, data including characteristics at baseline, operative details and in-hospital complication depends on data available from medical record review, which might had influence in comparing outcomes between both groups. Second, different influencing factors such as unmeasured patient characteristics and nursing care, which may affect outcomes of both groups, were not measured in this study. Third, Harris hip score and EQ-5D had not been assessed before the operation, which prevent us to analyze the changes of hip function and health-related quality of life before and after surgery. Forth, outcomes of dead patients were lost in our study, which might reduce the credibility of this study.

In conclusion, our results suggest that THA may be a preferred management option for active elderly patients over 75 years. The more extensive surgery of THA is not associated with higher in-hospital complication rate or mortality rate. These patients can benefit from THA in terms of hip function and quality of life.

## Data Availability

The data that support the findings of this study are available from the corresponding author upon reasonable request.

## Abbreviations

DFNF: Displaced femoral neck fracture
THA: Total hip arthroplasty
HA: Hemiarthroplasty
HHS: Harris hip score
EQ5D: EuroQol- 5 Dimensions
